# A90 ASSESSMENT OF A MULTI-COMPONENT QUALITY IMPROVEMENT INTERVENTION TO IMPROVE DIAGNOSTIC YIELD FROM ENDOSCOPIC ULTRASOUND-GUIDED FINE NEEDLE ASPIRATION BIOPSY OF SOLID MASS LESIONS

**DOI:** 10.1093/jcag/gwae059.090

**Published:** 2025-02-10

**Authors:** G Sandha, S Khan, P Mathura, L Puttagunta, S Girgis, A Thiesen, J Zhang, J Nilsson, S Wasilenko, S Zepeda-Gomez

**Affiliations:** University of Alberta College of Health Sciences, Edmonton, AB, Canada; University of Alberta College of Health Sciences, Edmonton, AB, Canada; University of Alberta College of Health Sciences, Edmonton, AB, Canada; University of Alberta College of Health Sciences, Edmonton, AB, Canada; University of Alberta College of Health Sciences, Edmonton, AB, Canada; University of Alberta College of Health Sciences, Edmonton, AB, Canada; Alberta Health Services, Edmonton, AB, Canada; University of Alberta College of Health Sciences, Edmonton, AB, Canada; University of Alberta College of Health Sciences, Edmonton, AB, Canada; University of Alberta College of Health Sciences, Edmonton, AB, Canada

## Abstract

**Background:**

A chart audit to review endoscopic ultrasound (EUS)-fine needle aspiration biopsy (FNAB) of solid mass lesions from 01/2022-12/2022 identified a diagnostic yield of 75%. To improve this, a quality improvement intervention including increasing the number of needle passes to 3/case, improving needle pass documentation in endoscopy reports, reducing the number of individuals making cytology slides, and using formalin as transport medium for cell block preparation instead of saline was developed and trialed for 9 months.

**Aims:**

To assess intervention impact on improving the diagnostic yield of EUS-FNAB of solid mass lesions.

**Methods:**

Three endoscopists were provided targeted education and a chart audit was completed for all patients undergoing EUS-FNAB of solid mass lesions from 01/2024-09/2024. Descriptive statistics were completed. Only a definite diagnosis, as confirmed on histological examination, was considered when calculating the diagnostic yield.

**Results:**

A total of 183 patients (112 M, 71 F), mean age 63±13 years (range 12-88 years), underwent 198 EUS-FNABs by 3 endoscopists (who made cytology slides, ensured transport medium and completed documentation). Pancreatic masses were the most common (122/198, 62%). A 22-gauge FNAB needle was used in 194/198 (98%) cases. A total of 420 needle passes were performed for 191 patient cases (mean 2.2/case) compared with mean of 1.9/case pre-intervention. Documentation improved, 7 cases (3.5%) did not have the number of needle passes specified compared with 51 cases (26%) pre-intervention. Tissue samples were transported in formalin for histology in 197 cases, on cytology slides in 165 cases, and in formalin for cell block preparation in 42 cases, similar to pre-intervention. Overall, a definite diagnosis was achieved in 156/198 cases (79%) compared with 149/200 (75%) in the pre-intervention year. Stratifying for needle passes, a definite diagnosis was achieved in 20/36 (56%), 69/87 (79%), 56/63 (89%), and 5/5 (100%) cases that had 1, 2, 3, and >3 needle passes, respectively. The number of passes was seen to independently impact diagnostic yield regardless of type of solid mass, endoscopist, type/size of needle used, and whether or not tissue was provided for cell block preparation.

**Conclusions:**

Although we did not reach our goal of 3 needle passes per case, documentation and the number of tissue samples transported in formalin improved. The results suggest 3 or more needle passes per case may improve diagnostic yield and attempts should be made to avoid performing single needle pass FNAB.

**Funding Agencies:**

None

